# Blood Pressure Response to Submaximal Exercise Test in Adults

**DOI:** 10.1155/2016/5607507

**Published:** 2016-09-15

**Authors:** Katarzyna Wielemborek-Musial, Katarzyna Szmigielska, Joanna Leszczynska, Anna Jegier

**Affiliations:** ^1^Department of Preventive Medicine, Medical University of Lodz, Pomorska 251, 92-513 Lodz, Poland; ^2^Department of Sports Medicine, Medical University of Lodz, Pomorska 251, 92-513 Lodz, Poland

## Abstract

*Background*. The assessment of blood pressure (BP) response during exercise test is an important diagnostic instrument in cardiovascular system evaluation. The study aim was to determine normal values of BP response to submaximal, multistage exercise test in healthy adults with regard to their age, gender, and workload.* Materials and Methods*. The study was conducted in randomly selected normotensive subjects (*n* = 1015), 512 females and 498 males, aged 18–64 years (mean age 42.1 ± 12.7 years) divided into five age groups. All subjects were clinically healthy with no chronic diseases diagnosed. Exercise stress tests were performed using Monark bicycle ergometer until a minimum of 85% of physical capacity was reached. BP was measured at rest and at peak of each exercise test stage.* Results*. The relations between BP, age, and workload during exercise test were determined by linear regression analysis and can be illustrated by the equations: systolic BP (mmHg) = 0.346 × load (W) + 135.76 for males and systolic BP (mmHg) = 0.103 × load (W) + 155.72 for females.* Conclusions*. Systolic BP increases significantly and proportionally to workload increase during exercise test in healthy adults. The relation can be described by linear equation which can be useful in diagnostics of cardiovascular diseases.

## 1. Introduction

Arterial hypertension is a serious medical, social, and economic problem in Poland. In WOBASZ Study conducted in Central Poland, hypertension was identified in 40% of males and 32% of females whereas more females than males were undergoing antihypertensive treatment (76% versus 61%, resp.) [1]. The study assessing the awareness of hypertension in hypertensive patients from Central Poland by Michalska et al. revealed that 79% of patients were unaware of optimal blood pressure range, 23.7% of the elderly subjects did not know the symptoms of hypertension, and 28.7% had poor awareness of hypertension therapy in the absence of symptoms [2]. Blood pressure response to physical exercise is an important diagnostic parameter assessed during submaximal physical exercise tests in diagnostic laboratories. Systolic blood pressure during dynamic, isotonic exercise is expected to rise according to the increasing workload. In clinically healthy patients, systolic arterial blood pressure increases during dynamic exercise and stabilizes after 2-3 minutes of exercise of a given intensity [3, 4]. Diastolic blood pressure in such conditions usually remains unchanged or may decrease insignificantly [3]. According to European and American experts, dynamic exercise of high intensity in normal conditions can cause the maximum value of systolic blood pressure to increase up to 250 mmHg and that of diastolic pressure up to 110 mmHg [5–8]. According to the recommendations by American College of Sports Medicine, the increase of dynamic exercise intensity by 1 MET should result in systolic blood pressure increase by 10 mmHg [9].

The relationship between the systolic blood pressure values and the exercise workload had already been determined with the linear regression by Heck et al.; however, the correlation was identified for the group composed mainly of athletes (90%) aged 10–71 years [10].

It has already been established that the excessive increase in blood pressure during exercise test on a cycle ergometer or a treadmill can be a prognostic factor for future development of hypertension or death due to cardiovascular complications [6, 11, 12]. Moreover, normotensive status maintained through pharmacological treatment proved to produce better results than optimal therapy in stroke prevention [13]. The paper presents new values of arterial blood pressure during exercise which can be of use in hypertension diagnostics as well as in hypertensive response to exercise for Polish population. Arterial blood pressure assessment during the exercise test concentrates mainly on the systolic blood pressure and as the measurement of the smallest risk of error, it is frequently employed in clinical practice [11, 12, 14].

The aim of the study was to define normal blood pressure response to multistage exercise test in healthy adults with reference to their age and gender at each workload stage.

## 2. Material and Methods

Randomly selected 2500 people were invited to take part in the study. 1121 of them took part and 106 were disqualified from the study. Eventually, the study included 1015 clinically healthy subjects. Adult participants of the study were randomly recruited based on the information provided by the Local Data Bank in Lodz that stores the data of 760 000 inhabitants. The distribution pattern of the draw was maintained. The attendance rate amounted to 44.8%. The study group was composed of 49.1% of males (*n* = 498) and 50.9% of females (*n* = 517). The study analysis included the subjects with an age range of 18–64 years (mean age 42.1 ± 12.7 years) divided into the following five age groups: 18–24 years (*n* = 147), 25–34 years (*n* = 154), 35–44 years (*n* = 186), 45–54 years (*n* = 330), and 55–64 years (*n* = 198).

On the basis of the subjects' history and their resting arterial blood pressure measured three times, they were qualified as the subjects with normal resting arterial blood pressure, on no medication, and without diagnosed chronic diseases such as arterial hypertension, ischemic heart disease, diabetes mellitus, kidney dysfunction, or heart failure. The subjects with myocardial infarction and cerebrovascular accident (CVA) in history were excluded from the study. The arterial blood pressure values <140/90 mmHg were considered as normal. The subjects were qualified for the exercise test after medical examination, resting arterial blood pressure measurement, echocardiography at rest, and anthropometric measurements taken (height, body weight, and BMI). The exercise tests were performed using the Monark bicycle ergometer Ergomedic 874 E where the study subjects were pedaling at 60 revolutions/minute with the workload increased every 3 minutes. Simultaneously, the heart bioelectrical function was being monitored by means of the cardiologic software Marquette CASE-16 and blood pressure was measured by Korotkov method. The arterial blood pressure measurements were performed at rest, at the left upper arm, and after 15 minutes' rest as well as in the last 45 seconds of each workload of exercise test while the subjects were still pedaling. The measurements were taken using the aneroid sphygmomanometer INCO-VERITAS S.A. which was calibrated and checked according to the recommendations of the European Society of Hypertension [5, 11, 15, 16]. INCO-VERITAS aneroid sphygmomanometers are certified medical devices used in accordance with European Union Law-adjusted Laws (Dz.U.Nr 126, 2001. Poz. 1379, 1380, 1382). The error of measurement for INCO-VERITAS aneroid sphygmomanometers was ±3 mmHg and its range of the measurement was between 0 and 300 mmHg. Standard cuffs with the arm circumference of 24–32 cm were used for arterial blood pressure measurement in adults. Bigger cuffs with the circumference reaching 32–42 cm were used for obese subjects. During exercise tests the study subjects were loaded with the consecutive, increasing workloads which lasted for about 3 minutes until achieving at least 85% of the maximum age-related heart rate or till any contraindications for the test continuation occurred. The contraindicating symptoms included chest discomfort, dizziness, severe exhaustion, pain in lower limbs, pathological abnormalities in echocardiography, or arterial blood pressure increase ≥250/115 mmHg [6]. The participants began exercise testing with a 1-2 minute's warm-up phase and then started cycling at 60 W with 30–60 W increment at each test stage according to HR achieved. The whole multistage test duration was 6–12 minutes and the rate of pedaling was 60 revolutions/min. The study was approved by the Bioethics Committee at the local Medical University.

Statistical analysis was performed with Statistica software (10.0 Star Soft). Values are expressed as arithmetic mean (*x*) and standard deviation (SD). A *p* value less than 0.05 was considered statistically significant. Linear relationships (first-order regression equations) with regard to one standard deviation were determined. ANOVA test and Kruskal-Wallis test were used for statistical analysis.

## 3. Results

Mean age of the study subjects was 42.1 ± 12.7 years. There were no statistically significant age differences between females and males (42.4 ± 12.1 years versus 41.7 ± 12.3 years, *p* = 0.1). The characteristics of the study subjects analyzed at rest as a whole group and according to the gender are presented in Table 1 and they include selected anthropometric parameters, resting heart rate, and systolic and diastolic arterial blood pressure. Males were characterized by statistically significantly greater height, body mass, higher BMI, and higher systolic and diastolic arterial blood pressure compared to females (*p* < 0.001). Females, in turn, showed a significantly higher resting heart rate in comparison to males (*p* < 0.00003) (Table 1).

Table 2 presents the values of the resting heart rate, the resting systolic and diastolic arterial blood pressure in relation to the gender and 5 age groups of the study subjects. Resting systolic and diastolic arterial blood pressure were statistically significantly higher in males on average by 6.1 ± 5.2 mmHg (*p* = 0.001) for the systolic pressure and by 2.7 ± 1.7 mmHg (*p* = 0.0001) for the diastolic pressure compared with females at the same age. Systolic and diastolic blood pressure increased statistically significantly with age of the study females and males (*p* = 0.0001) (Table 2).

Table 3 presents the characteristics of selected haemodynamic indices and exercise tolerance at peak workload in exercise testing (Table 3).

The increase of mean systolic blood pressure amounted to 44.4 ± 16.2 mmHg which was equal to 28% of the resting value. Female subjects completed their exercise test at the mean workload of 87.4 ± 36.9 (W) with the heart rate values of 159.2 ± 16.1 bpm, systolic arterial blood pressure 162.4 ± 25.6, and diastolic blood pressure 87.8 ± 11.1 mmHg, whereas males finished their exercise test at the mean workload of 122.2 ± 57.7 (W) with the heart rate values of 163.5 ± 13.2 bpm, systolic arterial blood pressure 173.7 ± 28.8, and diastolic blood pressure 89.8 ± 12.1 mmHg. Compared with the resting values, the diastolic arterial blood pressure during the exercise test increased statistically significantly (*p* = 0.0001) by mean 8.1 ± 1.3 mmHg. Exercise diastolic blood pressure values were statistically significantly higher in males than in females (*p* = 0.0001). The value of double product (peak HR·SBP) at peak workload of the exercise test was significantly higher in comparison with female subjects (*p* = 0.00001).

Table 4 shows the linear regression equations of exercise systolic arterial blood pressure in the analyzed age groups with regard to the gender and the workload (*x*). All the relationships were statistically significant (*r* from 0.4 to 0.6) (*p* < 0.0001).

**Figure 1:**
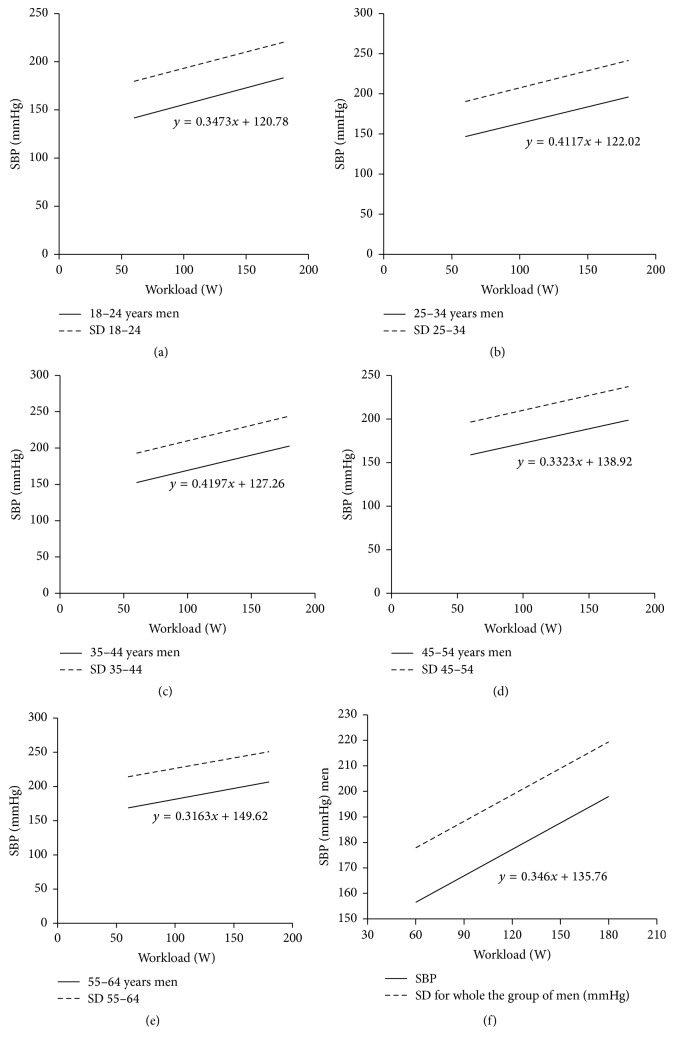
Regression line and two standards of deviation for linear relationship between exercise systolic blood pressure and exercise workload in all the males irrespective of age. Linear relationship between exercise systolic blood pressure and exercise workload in all the males irrespective of age and in particular age groups: (a) age group 18–24, (b) age group 25–34, (c) age group 35–44, (d) age group 45–54, (e) age group 55–64, and (f) all the study men.

**Figure 2:**
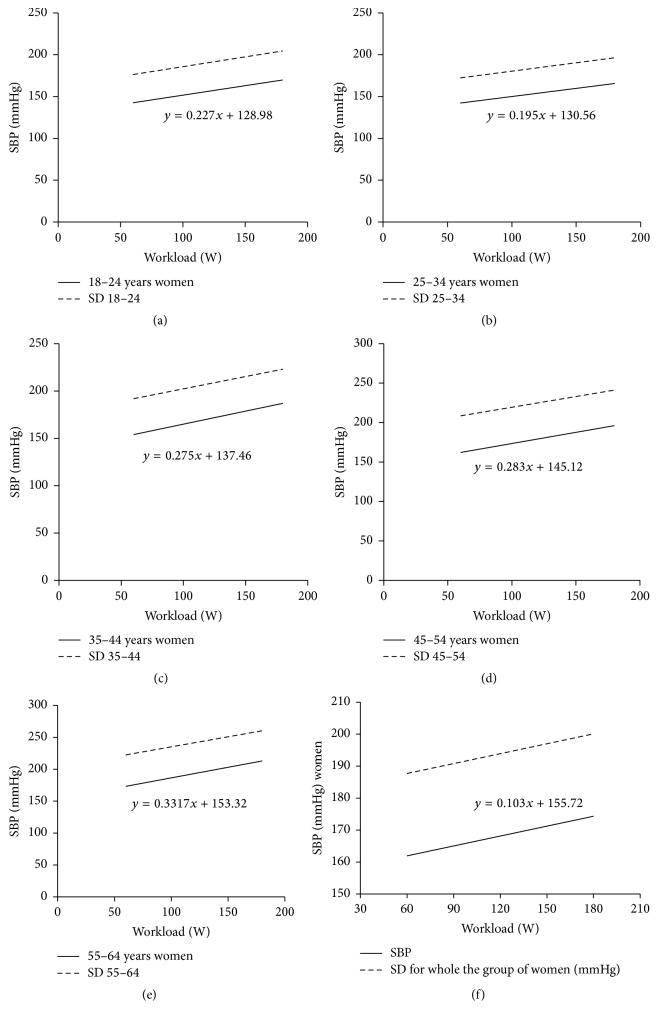
Regression line and two standards of deviation for linear relationship between exercise systolic blood pressure and exercise workload in all the females irrespective of age and in particular age groups: (a) age group 18–24, (b) age group 25–34, (c) age group 35–44, (d) age group 45–54, (e) age group 55–64, and (f) all the study women.

Figure 1 shows regression line and two standards of deviation for linear relationship between exercise systolic blood pressure and exercise workload in all the males irrespective of age. Figure 1 shows linear relationship between exercise systolic blood pressure and exercise workload in all the males irrespective of age and in particular age groups: Figure 1(a): age group 18–24; Figure 1(b): age group 25–34; Figure 1(c): age group 35–44; Figure 1(d): age group 45–54; Figure 1(e): age group 55–64; Figure 1(f): all the study men.  Figure 2 shows regression line and two standards of deviation for linear relationship between exercise systolic blood pressure and exercise workload in all the females irrespective of age and in particular age groups: Figure 2(a): age group 18–24; Figure 2(b): age group 25–34; Figure 2(c): age group 35–44; Figure 2(d): age group 45–54; Figure 2(e): age group 55–64; Figure 2(f): all the study women.

## 4. Discussion

In the present study, during single-bout, multistage exercise test on a cycle ergometer, systolic blood pressure increased significantly with the workload both in clinically healthy males and females. The relation between systolic blood pressure and workload can be expressed depending on sex, by the following formulae:

For the studied males,

(1) systolic  blood  pressure  mmHg=0.346×workloadW+135.76.

For the studied females,

(2) systolic  blood  pressure  mmHg=0.103×workload  W+155.72.

In clinical practice the cycle ergometer appears more applicable than the treadmill in exercise testing as it enables more stability for electrocardiography and more accuracy in blood pressure assessment [11, 17, 18]. Pressure response to exercise at a given workload was found to be more dynamic in healthy males than in females. Other studies also demonstrated that females younger than 50 years of age had lower blood pressure values both at rest and during exercise testing compared with males at similar age [19]. Clinical and epidemiological studies demonstrate that estrogens secreted in women in their perimenopausal period can play an important role in arterial blood pressure regulation [19].

Most studies where diagnostic and prognostic value of blood pressure measurements during exercise test were assessed were carried out on a cycle ergometer. Applying the cycle ergometer enables standardization of the procedure according to the test duration and workload determination, as in treadmill exercise testing. Some authors, however, suggest that the cycle ergometer also enables more accurate blood pressure assessment due to a better shoulder stabilization during measurements [11]. It has been reported in literature that noninvasive measurements of diastolic blood pressure could be inaccurate, particularly while using a mercury or aneroid sphygmomanometer, and are characterized by low measurement reproducibility [11]. This can result from the observation that Korotkov sound 5 is sometimes heard throughout the measurement period; that is, until point zero is achieved, as in healthy young people [6]. Therefore, the results of diastolic blood pressure measurements during exercise testing should be carefully evaluated, particularly when the observed changes are small [20]. Probably that is why data collected during physical exercise usually refer to the values of systolic pressure and such data are most frequently used in clinical practice [20].

Nearly 30 years ago Heck et al. studied the relation between workload and systolic blood pressure in about 3000 healthy subjects aged between 10 and 71 years, 90% of whom were sportsmen [10]. In the present study the mean values of systolic blood pressure at a given workload were found to be higher than those reported for the German athletes. This study also recorded statistically significant differences of exercise arterial blood pressure values between females and males.

According to Heck, the calculated systolic blood pressure value for a given age and workload should correspond to the mean value increased or reduced by 1SD for the study population [10]. The formula, however, is of limited applicability for Polish population. While diagnosing arterial hypertension, “white coat hypertension,” or hypertensive response to exercise it seems more functional to take into account only the mean value increased by 1SD.

Fletcher et al. reported the results of a similar study that evaluated the blood pressure response to various workloads [6]. Their findings concerned the pressure values at peak submaximal exercise test on a treadmill performed in a group of more than 700 healthy males aged between 25 and 54 years. In that study, a double maximal product (heart rate × systolic blood pressure) was calculated and presented as percentiles only for the highest workload [6]. In 2000, Jager proposed formulae for calculating the heart rate and the systolic blood pressure as a function of workload during dynamic exercise [21]. However, the formulae included the sum of systolic and diastolic blood pressure values which is of little clinical application [21].

In the present study peak values of the systolic blood pressure during multistage exercise test did not exceed 190 ± 20 mmHg in women and 210 ± 20 mmHg in men [8, 12, 21, 22]. The dependence of BP response on age was tested using *r*-Pearson test and test for two regression coefficients. The dependence can be described as a linear relationship. The slope angle between the line reflecting systolic blood pressure values during exercise and the axis corresponding to workload values during exercise was larger in males than in females. The comparison of regression coefficients did not reveal statistically significant differences between systolic blood pressure values during exercise in the analyzed age groups. Systolic pressure increases with dynamic workload as a result of increased stroke volume, whereas diastolic blood pressure usually remains unchanged [11]. An excessive increase in systolic blood pressure during exercise testing can be predictive of the development of arterial hypertension in the future [21] and it can be associated with the development of ischemic heart disease confirmed by angiography [6, 21], whereas an inadequate increase in systolic blood pressure or its decrease during exercise testing can result from for example, severe dysfunction of the left ventricle, or heart muscle ischemia [6, 21].

The presented ranges of blood pressure values during exercise testing might be useful in clinical practice while diagnosing hypertension and they could facilitate the identification of patients with the hypertensive response to exercise [23–29]. The strength of the present study is that it was performed in a relatively large, randomly selected population of males and females and its methods can easily be applied and repeated. Its weaknesses include the fact that workloads achieved during exercise testing were not maximal. It should be noted, however, that reaching a minimum of 85% of maximum age-related heart rate is a commonly used method by many laboratories in practice.

## 5. Summary of the Results and Conclusions

(1) The relationship between systolic arterial blood pressure and the workload during exercise test in adults can be described by the following equations:
 For adult males aged 18–64 years,
  (3) exercise  systolic  blood  pressure  mmHg=0.346×workload  W+135.76.
 For adult females aged 18–64 years,
  (4) exercise  systolic  blood  pressure  mmHg=0.103×workload  W+155.72.
(2) The values can be applied for monitoring normal and hypertensive response to exercise, particularly in prevention of cardiovascular diseases.

## Figures and Tables

**Table 1:** Characteristics of the study subjects, selected anthropometric and cardiological parameters.

Parameter	Study group *n* = 1015 *x* ± SD	Males *n* = 498 *x* ± SD	Females *n* = 517 *x* ± SD	*p* Males versus females
Age (years)	42.1 ± 12.7	41.7 ± 12.3	42.4 ± 12.1	*p* = 0.1
Height (cm)	169 ± 9.5	176.5 ± 6.5	162.9 ± 6.3	*p* = 0.001
Body mass (kg)	75.1 ± 15.1	83.3 ± 13.0	67.1 ± 12.7	*p* = 0.00001
BMI (kg/m^2^)	26.2 ± 5.2	27.0 ± 5.5	25.3 ± 4.9	*p* = 0.00001
HR rest (bpm)	84.6 ± 14.5	82.6 ± 14.4	86.5 ± 13.9	*p* = 0.000003
SBP rest (mmHg)	123.7 ± 11.0	124.9 ± 12.1	122.4 ± 9.8	*p* = 0.00001
DBP rest (mmHg)	80.6 ± 5.7	81.1 ± 7.3	79.8 ± 2.0	*p* = 0.00001

**Table 2:** Mean values of resting heart rate and systolic arterial blood pressure (SBP) and diastolic (DBP) arterial blood pressure in relation to gender and age.

Age group	Parameters					
	HR rest (bpm)		SBP rest (mmHg)		DBP rest (mmHg)	
	*x* ± SD		*x* ± SD		*x* ± SD	
	Males	Females	Males	Females	Males	Females
	*n* = 498	*n* = 517	*n* = 498	*n* = 517	*n* = 498	*n* = 517
18–24 years *n* = 147	85.1 ± 15.1^*∗*^ *n* = 67	91.1 ± 16.3 *n* = 80	123.8 ± 12.0^*∗*^ *n* = 67	114.9 ± 11.2 *n* = 80	79.8 ± 8.7	73.6 ± 8.8

25–34 years *n* = 154	84.4 ± 13.7^*∗*^ *n* = 81	88.9 ± 15.6 *n* = 73	123.3 ± 14.3^*∗*^ *n* = 81	113.2 ± 11.5 *n* = 73	80.6 ± 7.5	74.9 ± 9.0

35–44 years *n* = 186	84 ± 15.3^*∗*^ *n* = 110	86.1 ± 12.4 *n* = 76	124.3 ± 11.3^*∗*^ *n* = 110	118.2 ± 15.3 *n* = 76	81.3 ± 7.3	78.4 ± 9.8

45–54 years *n* = 330	82.6 ± 14.3^*∗*^ *n* = 150	83.8 ± 13.3 *n* = 180	125.7 ± 12.9 *n* = 150	122.8 ± 13.6 *n* = 180	82.6 ± 6.8	80.3 ± 8.4

55–64 years *n* = 198	77.8 ± 13^*∗*^ *n* = 90	86.4 ± 13.6 *n* = 108	127.3 ± 11.2 *n* = 90	126.0 ± 11.4 *n* = 108	82.3 ± 6.7	81.2 ± 7.2

^*∗*^ *p* < 0.05 statistical significance of males versus females differences.

**Table 3:** The characteristics of selected hemodynamic indices and exercise tolerance at peak workload during exercise test in study subjects (*n* = 1015).

Exercise data *n* = 1015	Males *n* = 498 *x* ± SD	Females *n* = 517 *x* ± SD	*p* Males versus females
Peak workload (W)	122.2 ± 57.7	87.4 ± 36.9	*p* = 0.001
Peak HR (bpm)	163.5 ± 13.2	159.2 ± 16.1	*p* = 0.00001
ΔHR (peak HR − HR rest)	46.1 ± 9.2	51.1 ± 6.9	*p* = 0.000003
Peak SBP (mmHg)	173.7 ± 28.8	162.4 ± 25.6	*p* = 0.00001
ΔSBP (peak SBP − SBP rest)	+48.8 ± 16.7	+40 ± 15.8	*p* = 0.00001
Peak DBP (mmHg)	89.8 ± 12.1	87.8 ± 11.1	*p* = 0.00001
ΔDBP (peak DBP − DBP rest)	+8.7 ± 4.8	+8.0 ± 4.0	*p* = 0.00001
Double product (peak HR × peak SBP bpm *∗* mmHg) RPP × 10^−2^	284 ± 3.8	258 ± 4.1	*p* = 0.00001

**Table 4:** Linear regression equations of exercise systolic arterial blood pressure (SBP mmHg) in the analyzed age groups with regard to their gender and the workload in watts (*x*).

Age group	Males *n* = 498	±SD	Females *n* = 517	±SD	Test for two regression coefficients
Total *n* = 1015	*y* = 0.346*x* + 135.76 *r* = 0.6; *p* < 0.0001	22.9	*y* = 0.103*x* + 155.72 *r* = 0.4; *p* < 0.0001	23.6	*p* = 0.000001

18–24 years *n* = 147	*y* = 0.3473*x* + 120.78 *n* = 67 *r* = 0.5; *p* = 0.00001	20.1	*y* = 0.227*x* + 128.98 *n* = 80 *r* = 0.5; *p* = 0.00001	17.2	*p* > 0.05

25–34 years *n* = 154	*y* = 0.4117*x* + 122.02 *n* = 81 *r* = 0.5; *p* = 0.00001	22.9	*y* = 0.195*x* + 130.56 *n* = 73 *r* = 0.4; *p* = 0.00001	15.8	*p* > 0.05

35–44 years *n* = 186	*y* = 0.4197*x* + 127.26 *n* = 110 *r* = 0.5; *p* = 0.00001	22.5	*y* = 0.275*x* + 137.46 *n* = 76 *r* = 0.4; *p* = 0.00001	17.8	*p* > 0.05

45–54 years *n* = 330	*y* = 0.3326*x* + 138.92 *n* = 150 *r* = 0.4; *p* = 0.00001	21.7	*y* = 0.283*x* + 145.12 *n* = 180 *r* = 0.5; *p* = 0.00001	24.4	*p* > 0.05

55–64 years *n* = 198	*y* = 0.3163*x* + 149.62 *n* = 90 *r* = 0.4; *p* = 0.00001	24.1	*y* = 0.3317*x* + 153.32 *n* = 108 *r* = 0.4; *p* = 0.00001	23.2	*p* > 0.05
